# Dopaminergic Modulation of Sensory Attenuation in Parkinson's Disease: Is There an Underlying Modulation of Beta Power?

**DOI:** 10.3389/fneur.2019.01001

**Published:** 2019-09-18

**Authors:** Antonella Macerollo, Patricia Limousin, Prasad Korlipara, Tom Foltynie, Mark J. Edwards, James Kilner

**Affiliations:** ^1^The Walton Centre NHS Foundation Trust, Liverpool, United Kingdom; ^2^School of Psychology, Faculty of Health and Life Sciences, University of Liverpool, Liverpool, United Kingdom; ^3^National Hospital for Neurology and Neurosurgery, London, United Kingdom; ^4^Institute of Neurology, University College of London, London, United Kingdom; ^5^Department of Neurology, St George's University of London, London, United Kingdom

**Keywords:** Parkinson's disease, sensory attenuation, beta power, bradykinesia, motor symptoms

## Abstract

**Background and Aims:** Pathological high amplitude of beta oscillations is thought as the underlying mechanism of motor symptoms in Parkinson's disease (PD), in particular with regard to bradykinesia. In addition, abnormality in a neurophysiological phenomenon labeled sensory attenuation has been found in patients with PD. The current study explored the hypothesis that the abnormal sensory attenuation has a causal link with the typical abnormality in beta oscillations in PD.

**Methods:** The study tested sixteen right-handed patients with a diagnosis of PD and 22 healthy participants, which were matched by age and gender. Somatosensory evoked potentials were elicited through electrical stimulation of the median nerve at the wrist. Electrical activity was recorded at the scalp using a 128 channels EEG. Somatosensory evoked potentials were recorded in 2 conditions: at rest and at the onset of a voluntary movement, which was a self-paced abduction movement of the right thumb.

**Results:** Healthy participants showed a reduction of the N20-P25 amplitude at the onset of the right thumb abduction compared to the rest condition (*P* < 0.05). When patients were OFF medication, they showed mild reduction of the N20-P25 component at movement onset (*P* < 0.05). On the contrary, they did show greater attenuation of the N20-P25 component at the onset of movement compared to the rest condition when ON medication (*P* < 0.05). There was no significant evidence of a link between the degree of sensory attenuation and the change in beta oscillations in our cohort of patients.

**Conclusion:** These results confirmed a significant link between dopaminergic modulation and sensory attenuation. However, the sensory attenuation and beta oscillations were found as two independent phenomena.

## Introduction

Several studies have showed that sensory afferents are reduced prior to and during movement (1–4). This phenomenon is denominated sensory attenuation (SA) or sensory gating.

A recent theoretical framework, labeled active inference, proposed that SA prior to and during active movement is an essential mechanism that allow to move (5).

This model of movement initiation hypothesizes that the brain needs to perceive when sensory information is uncertain and must down weight these external sensations to top-down predictions. In line with this hypothesis, the movement initiation is a consequence of fulfilling prior expectations about proprioceptive sensations. In other words, the movements are allowed by the transition from one sensory state to another. According to this model, an impairment to correctly initiate or maintain a voluntary movement might be due to an abnormality of SA (6).

It is still unknown if the pathophysiology of bradykinesia in Parkinson's disease (PD) is due to a deficit in SA. The latter is thought to be linked with pathology in reducing the precision of the somatosensory expectations (6).

The SA can be tested in two different fields: physiological and perceptual (7). The neurophysiological measure of SA is represented as a reduction in amplitude of somatosensory evoked potentials (SEPs) components at the onset of a voluntary movement compared with a rest condition (7).

SA is expected to be reduced in PD and improved with medical treatment. Indeed, SA prior to and during movement (as measured by a decrease in the amplitude of N20-P25 component of SEPs elicited by median nerve stimulation) has been found significantly reduced in PD patients OFF medication (8). Moreover, SA was normalized by dopaminergic medication (8). Of note, an attenuation of the N20-P25 component at the onset of voluntary movements in healthy participants (8).

This study aimed to replicate results of the previous study (8) in a completely naïve group of PD patients. The prediction was an interaction in the SEPs amplitude between group and time with the SEPs being more greatly attenuated in healthy controls at the onset of active movement than the patients' group in OFF state. Furthermore, it was predicted that there would not be any significant differences in SA between healthy participants and patients ON medication.

A second aim was to test whether SEPs attenuation was modulated as a function of disease and voluntary movement. In other words, it was tested if there was a correlation between the difference in N20-P25 amplitude between baseline and movement condition with measurements of bradykinesia using the Unified Parkinson's Disease Rating scale (UPDRS) (9) as well as parametric measures of the tapping through a cybernetic glove.

The prediction was that SEPs attenuation would correlate with movement such that the faster and more vigorous movements would be positively correlated with the degree of the SA. It was predicted that across subjects the lower (better) the UPDRS scores and the less slowing and decrement in amplitude of tapping measured by cyberglove, the greater the SA measured at movement onset. These results would be further support of the pathophysiological role of SA in the contest of bradykinesia.

Notably, the active inference theory makes more detailed predictions (10). It predicts that SA will be driven by a change in the precision of the sensory expectation, with lower precision leading to greater SEPs attenuation.

The second part of the study explored if SA modulations would be correlated with modulation in beta power in the sensorimotor cortex, which decrease prior to and during movement (11, 12).

Recently, Tan et al. (13) proposed a novel theory based on the functional role of sensorimotor post-movement beta synchronization (PMBS). This theory linked theoretical models of motor control related to a phenomenon called uncertainty and neurophysiological measures of sensorimotor activity. Indeed, voluntary movements stimulate peripheral sensory receptors providing sensory feedback of the movement action.

Adams et al. (10) tested a model hypothesizing that the predicted sensory consequences of a movement are compared to the actual sensory input. These authors calculated the prediction error by the difference between the predicted and actual sensory input. The prediction error is a measure used to make the forward model able to perform more accurate future predictions. Estimations of the uncertainty in the motor prediction and the uncertainty of the actual sensory input are required to calculate the importance of any prediction errors (14).

Tan et al. (13) have proposed modalities to manipulate the uncertainty. In addition, these authors predicted that PMBS would be correlate with the uncertainty rather than with the movement error. The PMBS amplitude over sensorimotor cortex was found to be characterized by negative correlation with the variable of uncertainty. Consequentially, this result supports a novel functional role of PMBS linking beta oscillations to the uncertainty of the parameters underlying the motor control. In other words, sensorimotor beta oscillatory power might be the neurophysiological mechanism allowing to estimate of uncertainty or causally modulating the uncertainty.

Palmer et al. (15) highlighted that this potential correlation between PMBS and sensory uncertainty might mean that beta oscillatory activity is a potential candidate for this sensory gating phenomenon. If beta oscillations modulation would be correlated with the time course of SEPs attenuation, this would be evidence that there might be a potential link between beta oscillatory activity and SA.

This finding is particularly relevant for the application of this theoretical account to explain akinesia and bradykinesia. In PD beta oscillations in the motor network and in the STN are higher during rest. Consequentially, pathological higher beta oscillations have been causally implicated in movement impairment rather than being just an epiphenomenon of the diseased state (16).

One theory therefore is that patients with PD have high sensory precision such that when they decide to move, they cannot attenuate this precision enough to allow the influence of top-down proprioceptive predictions to supersede. This theory is supported by our study, which has demonstrated decreased SA in patients diagnosed with PD compared to age-matched healthy controls (8, 17). Furthermore, dopaminergic treatment acted to normalize SA in PD patients, which suggests this may be one of the mechanisms which can explain the improvement in motor symptoms under this class of medication (8).

Here, it was tested if the specific time course of the SA is correlated with modulations in beta power during movement execution. The prediction was that modulations in beta power will be positively correlated with the time course of SEPs modulation. If this is the case, it will establish a statistical dependency between beta power and SA.

## Methods

Sixteen patients diagnosed with idiopathic PD (10 males, 6 females; mean age, 68 years; range, 52–79 years; Table 1) and 22 age and sex matched healthy participants (14 males, 8 females; mean age, 67 years; range, 50–80 years) were involved in the study. Control subjects were recruited from a pool of healthy subjects of the University College of London. This group of participants were not diagnosed with any medical disorder and they were not on medication.

**Table 1:** Clinical and demographic characteristics of patients with Parkinson disease (Mo, months; y, years; UPDRS, Unified Parkinson's Disease Rating Scale; SD, standard deviation; L, L-DOPA; D, Dopamine agonist).

	**Age** **(y)**	**Gender**	**Disease duration** **(y)**	**Motor** **UPDRS** **upper limbs bradykinesia items** **OFF state**	**Motor** **UPDRS** **upper limbs bradykinesia items** **ON state**	**Treatments**
1	72	M	11	11	6	L
2	75	F	4	9	5	L
3	61	M	2	6	3	L
4	75	M	5	11	5	L
5	77	F	10	9	5	L
6	68	F	4	6	3	L
7	56	M	4	8	3	L
8	70	F	6	6	3	L+D
9	69	M	6	9	4	L+D
10	79	F	12	10	6	L+D
11	68	F	10	12	6	L+D
12	52	M	10	12	6	L+D
13	62	M	3	8	3	L+D
14	68	M	8	12	9	L+D
15	72	M	5	8	3	L+D
16	68	M	5	8	3	L+D
Mean ± SD	68.1 ± 6.9	F8/M12	6.5 ± 2.9	9 ± 2	4.3 ± 1.7	

PD patients were recruited from the Movement Disorders Clinics at the National Hospital of Neurology and Neurosurgery.

Idiopathic PD was diagnosed according to the UK PD Society Brain Bank criteria (18) and further confirmed by abnormal dopamine transporter SPECT in all patients.

Participants did not have disabling tremor. None of the patients had cognitive decline. PD patients were on levodopa medication and/or on dopaminagonists.

Participants were right-handed.

The study was approved by the East of Scotland Research Ethics Service. Written informed consent was obtained from all participants.

Clinical disease severity was assessed with the motor section (items 3.1–3.18) of the UPDRS (9). The clinical assessment was performed in the ON as well as OFF state in each patient.

The amplitude and the frequency of a minute right hand tapping test with the Cyber Glove was recorded in both pharmacological states.

To reach the OFF state, patients were required not to take levodopa for at least 12 h and dopamine-agonists for at least 24 h prior to testing. Patients were assessed in the ON state 1 h after taking levodopa or 2 h after taking dopamine agonists (Table 1).

### Procedure and Experimental Design

Participants were seated in a comfortable armchair with hands relaxed on the armrest of the chair and their eyes closed. Two electrodes were placed on the surface of the wrist. The anode was placed over the median nerve at the wrist and the cathode 2 cm proximal to the anode. SEPs were elicited by electrical stimulation of the median nerve at the right wrist using a constant current square-wave pulse (0.2 ms duration). The intensity of the stimulation at threshold (slight thumb twitch) was identified and then increased by 1 mA to produce a definite thumb twitch. The intensity remained the same throughout the experiment.

Electrical activity was recorded at the scalp using a 128 channels Biosemi ActiveTwo AD-box EEG. EEG was recorded at a sampling rate of 2,048 Hz.

Surface electromyography (EMG) of the right abductor pollicis brevis (APB) was monitored simultaneously.

SEPs were recorded in three conditions in a single session.

In the baseline condition, the subjects were relaxed and instructed not to react to the stimulus. The frequency of the median nerve stimulation was 0.5 Hz. Subjects received 500 stimulations in this condition.

In the movement condition, subjects were instructed to make a self-paced abduction movement of the right thumb with a frequency of around a movement every second. At the onset of the movement, the median nerve stimulus was automatically triggered. The frequency of movements was recorded. Participants made 500 thumb abductions.

In the rest condition, the subjects were relaxed and instructed not to react to the stimulus. In distinction to the baseline condition here the median nerve stimulations were given at precisely the same times as the self-paced movements recorded from the movement condition.

### Data Analysis

#### Measure of SEPs Components and SA

EEG data analyses were performed in MATLAB 2013b (Math Works, Natick, MA, USA) using the software Statistical Parametric Mapping (SPM12, Wellcome Department of Imaging Neuroscience, London, UK).

The SEPs produced at movement onset has previously been employed to assess the degree of SA during active movement. Indeed, SEPs elicited by stimulation at this time point is not confounded by any possible effect of the afferent signal produced by the movement. The initial analysis was focused on modulations in the SEPs components, specifically the amplitude of the N20 and P25 as a function of group (PD patients ON medications, PD patients OFF medication and healthy participants). The peak-to-peak amplitude of the N20-P25 component was measured for each participant. EEG data were analyzed in SPM12.

The offline data were high-passed filtered at 0.1 Hz and, then, epoched to the time of the onset of the median nerve stimulation taking the 100 ms before stimulation and 250 ms after the stimulation. The data were baseline corrected by subtracting the average of the signal in a window from 20 to 5 ms prior to median nerve stimulation.

Artifacts exceeding 100 mV were manually rejected.

SEPs were averaged across the 500 trials of each condition. The baseline condition was the reference to select the appropriate channels to see N20 and P25. The electrodes over sensorimotor cortices were selected based on electrodes contralateral to the stimulated wrist that showed a negative peak at around 20 ms and a positive peak around 25–35 ms after the stimulus.

Then, the data from the selected channels were averaged and the amplitude and the time data points of N20 and P25 were measured. These electrodes and time points were used to calculate the amplitude of the N20 and P25 in the other two conditions—rest condition and movement condition. Note that the choice of electrodes and time points from an independent condition removed selection bias in the two experimental conditions of interest.

The SA was measured through the difference in the absolute amplitude of the peak N20-P25 between the rest and movement onset conditions was calculated.

### Analysis of Parametric Measures of Tapping and Quantification of Bradykinesia

The finger-tapping performed using the cyber glove was recorded through a Matlab script. The amplitude and the frequency of each tapping movement in a minute of interval time were calculated using Welch's power spectral density estimate of the time series of the tapping as recorded by the CyberGlove. The data were then averaged, and the peak amplitude and frequency at the peak amplitude of the tapping was taken for each pharmacological state of each patient. These were the parametric measures of tapping.

The regression analysis between SA and parametric measures of tapping was performed to test the hypothesis of a correlation between dopaminergic modulation of SA and dopaminergic improvement of bradykinesia.

### Analysis of Beta Power in Movement and Rest Condition

In healthy subjects, power in beta oscillations is expected to be attenuated prior to the thumb movement and augmented once the movement has ended (12).

After raw data conversion, EEG data were re-referenced by subtracting the average signal from two external electrodes attached to the subjects' earlobes from the signal from each EEG electrode. Data were high pass (0.1 Hz) filtered and down-sampled to 400 Hz.

A trigger was sent to the EEG system at the time of every median nerve stimulus. The data were epoched to the time of median nerve stimulation, taking the 1,000 ms before the onset and 1,000 ms after.

The different experimental blocks were merged into a single file.

For the time–frequency analysis, the power of the EEG signal at each frequency from 1 to 99 Hz in steps of 2 was estimated using the Morlet spectral estimation in SPM. The data were rescaled using a logarithmic transformation and averaged across all trials.

The time–frequency data were averaged over the same electrode channels selected for the SEPs analysis on the scalp map to investigate the modulation of beta power in each condition (rest and movement) for each subject and in each pharmacological state for each patient.

Subsequently, the time-frequency images for the rest condition for each subject were averaged across all subjects and three time' windows. The latter corresponded to the three phases of beta oscillations modulation with median nerve stimulation in the rest condition and were calculated as background (between 180 and 625 ms before the stimulus), suppression (between 165 and 378 ms after stimulus) and rebound (between 535 and 980 ms).

The beta power, obtained by averaging over the frequency of 15–25 Hz, was then averaged over each selected time window across subjects of each group to have a value of beta power for each time window per group per condition. Subsequently, a value of beta power modulation for each group and each time window was obtained through a subtraction of beta power value between rest and movement condition.

The value of beta power modulation was then regressed against the amplitude of SA per group per time window.

Finally, a regression analysis was performed between the amplitude of beta power and amplitude of SEPs for each group per time window per condition.

## Results

### SEPs Components and SA

**Figure 1:**
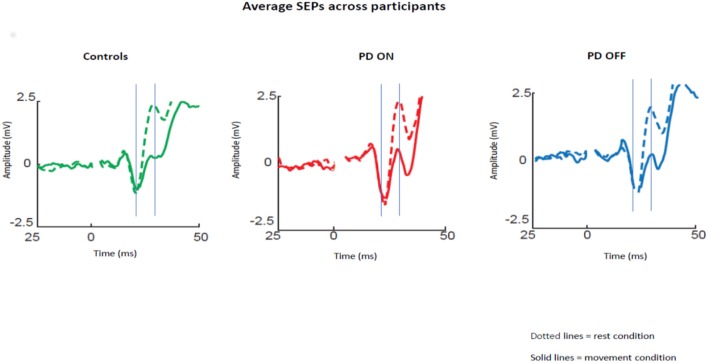
Average SEPs across participants recorded from the left somatosensory cortex for PD patients OFF medication, ON medication, and control subjects. Solid lines show data for median nerve stimulation given at movement onset and dotted lines during baseline. The gray lines show the mean time of the peaks of the N20 and P25 components.

The averaged SEPs over our ROI (channels over the somatosensory cortex) across participants for PD patients OFF medication, ON medication, and control subjects are shown in Figure 1.

Repeated measures ANOVA with the group (ON vs. OFF) and condition (rest vs. movement) as factors showed a significant effect of the condition [*p* < 0.05; *F*_(1,30)_ = 39.46; Eta^2^ = 0.537] and a significant interaction between condition and pharmacological state [*p* < 0.05; *F*_(1,30)_ = 6.33; Eta^2^ = 0.157]. *Post-hoc* pairwise comparisons revealed a significant difference between N20-P25 peak to peak amplitude between the rest condition and movement condition [*p* < 0.05; *t*_(30)_ = 5.85].

**Figure 2:**
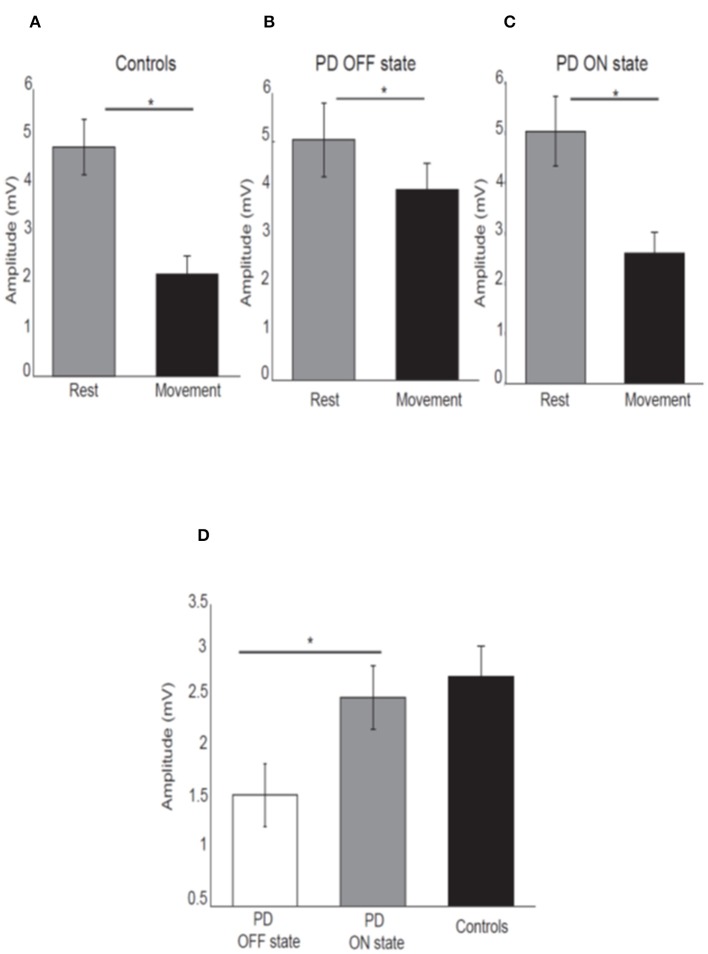
Mean amplitude of the N20-P25 component for each condition for control subjects **(A)**, PD patients OFF medication **(B)**, ON medication **(C)**. Error bars show standard error of the means. Mean difference of the N20-P25 amplitude between rest condition and movement condition in PD patients OFF medication, ON medication and controls **(D)**. *p < 0.05.

As expected, healthy participants showed attenuation of the N20-P25 amplitude at movement onset (2.13 ± 1.87) compared to the rest condition (4.8 ± 2.84) [*P* < 0.05; *t*_(21)_ = 7.45, Figure 2A].

PD patients OFF medication showed mild attenuation of the N20-P25 component at movement onset (3.99 ± 2.31) compared to rest condition (5.03 ± 3.29) [*P* < 0.05; *t*_(15)_ = 2.52; Figure 2B]. This group showed greater attenuation of the N20-P25 component at the onset of movement (2.59 ± 1.79) compared to the rest condition (5.02 ± 2.94) when ON medication [*P* < 0.05; *t*_(15)_ = 5.95; Figure 2C].

There was a significant difference in the amplitude of N20-P25 peak during the movement condition between OFF state (3.99 ± 2.31) and ON state (2.59 ± 1.79) [*p* = < 0.05; *t*_(15)_ = 3.32] with a smaller amplitude in the ON state.

There was no difference in the N20-P25 amplitude during the rest condition between OFF state (5.03 ± 3.29) and ON state (5.02 ± 2.94) [*p* ≥ 0.05; *t*_(15)_ = 0.017].

The SA (defined as difference in the amplitude of N20-P25 peak between rest condition and movement condition) showed a significant difference between OFF (1.29 ± 1.55) and ON state (2.42 ± 1.55) in PD patients [*p* ≤ 0.05; *t*_(15)_ = −3.28] with greater SA in ON state (Figure 2D).

There was no difference in the SA between PD patients in ON state (2.42 ± 1.55) and healthy subjects (2.74 ± 1.61) [*p* ≥ 0.05, *t*_(36)_ = −0.46] (Figure 2D).

**Figure 3:**
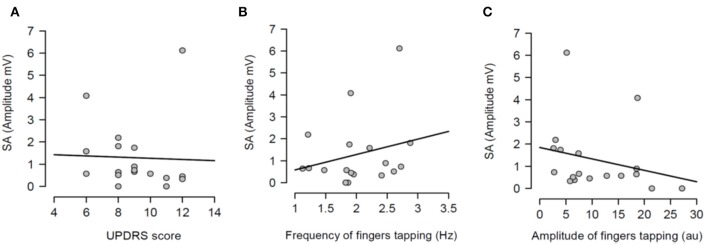
Regression analysis between sensory attenuation (SA) and measures of bradykinesia [UPDRS score **(A)**, frequency of fingers tapping **(B)** and amplitude of fingers tapping **(C)**] in PD patients in OFF state.

**Figure 4:**
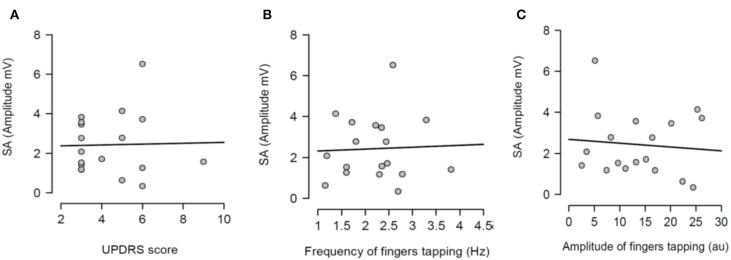
Regression analysis between sensory attenuation (SA) and measures of bradykinesia [UPDRS score **(A)**, frequency of fingers tapping **(B)** and amplitude of fingers tapping **(C)**] in PD patients in ON state.

Having shown that SA was modulated by dopaminergic treatment and that SA was significantly attenuated in PD patients ON medication, it was tested if the severity of right arm bradykinesia was correlated with the degree of SA. In this regard, there was no statistically significant correlation between SA and UPDRS scores (*R*^2^ = 0.001, *p* = 0.893 OFF medication (Figure 3A) and *R*^2^ = 0.001, *p* = 0.924 ON medication (Figure 4A) as well as between SA and frequency of the fingers tapping (*R*^2^ = 0.059, *p* = 0.330 OFF medication (Figure 3B) and *R*^2^ = 0.002, *p* = 0.867 ON medication (Figure 4B) or amplitude of the fingers tapping (*R*^2^ = 0.06, *p* = 0.323 OFF medication (Figure 3C) and *R*^2^ = 0.008, *p* = 0.718 ON medication (Figure 4C).

**Figure 5:**
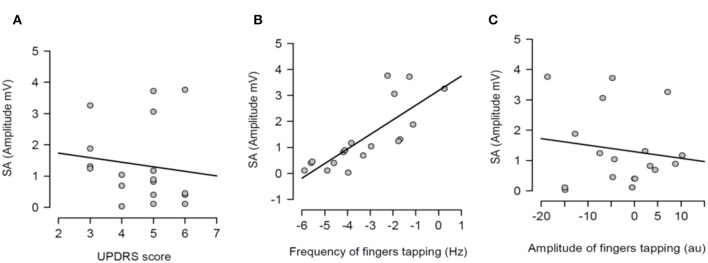
Regression analysis between dopaminergic changes of sensory attenuation (SA) and dopaminergic changes of each measure of bradykinesia [UPDRS score **(A)**, frequency of fingers tapping **(B)** and amplitude of fingers tapping **(C)**]. The dopaminergic changes of each variable were calculated through the difference between OFF and ON values for each variable.

After having tested the hypothesis of a potential correlation between SA and each measure of bradykinesia in the individual pharmacological state, a potential correlation between the dopaminergic modulation of SA and the dopaminergic modulation of each measure of bradykinesia was investigated. In other words, it was tested if there was a correlation between SA changes between OFF and ON states and changes of each measure of bradykinesia between OFF and ON states. There was no statistically significant correlation between dopaminergic modulation of SA and changes of UPDRS scores (*R*^2^ = 0.016, *p* = 0.616) (Figure 5A). There was a significant correlation between dopaminergic modulation of SA and changes of frequency of the fingers tapping (*R*^2^ = 0.623, p <0.001) (Figure 5B). However, there was not significant correlation with the amplitude of the finger tapping at this frequency (*R*^2^ = 0.021, *p* = 0.562) (Figure 5C).

### Beta Oscillations Modulation

Having demonstrated that there was a modulation of SEPs over condition, the second aim was to test if the SA was correlated with modulations in beta oscillations over the sensorimotor cortex.

Firstly, it was tested the hypothesis that healthy controls and PD patients showed a modulation of beta power as function of time in each experimental condition. The prediction was to find power in beta oscillations attenuated prior to the thumb movement and a rebound at the end of the movement. After averaging the time-frequency images across subjects for each group, the changes of the beta power spectrum (interval of frequency at 15–30 Hz) as function of time in each condition were showed. Beta power was clearly evident prior to movement in the baseline period, suppressed in the motor preparation and execution period and, finally, rebounded at the end of the thumb movement.

**Figure 6:**
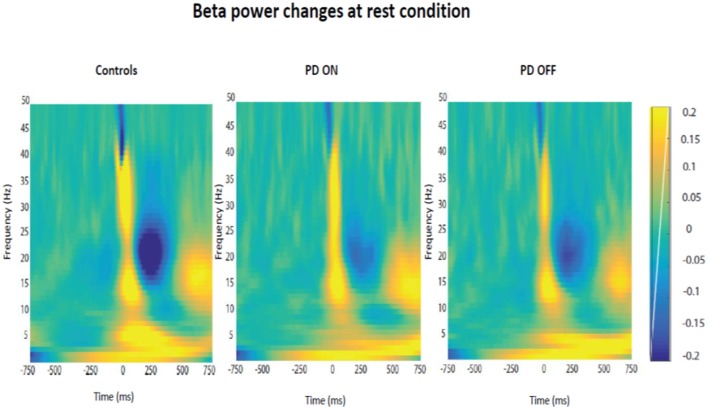
Modulation of beta power changes as function of time at rest in each group of participants (PD ON, PD OFF, Healthy Subjects).

The modulation of beta oscillations in the rest condition averaged across subjects for each group is showed in the Figure 6.

Following the qualitative analysis, a quantitative analysis of the beta oscillations was performed in three time' windows selected as explained in the methods section. The three times windows corresponded to the three phases of beta oscillations modulation calculated as background (between 180 and 625 ms before the stimulus), suppression (between 165 and 378 ms after stimulus) and rebound (between 535 and 980 ms).

**Figure 7:**
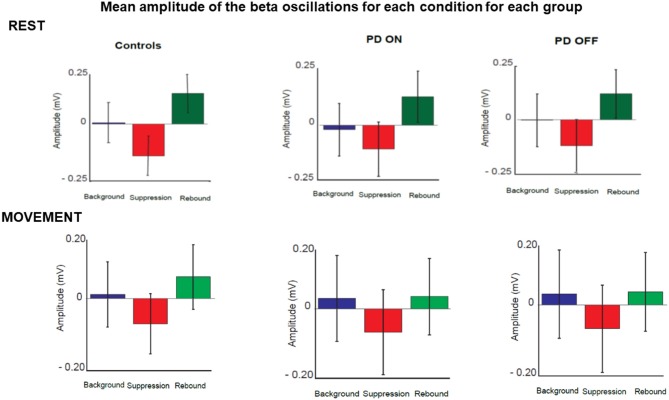
Mean amplitude of beta oscillations for each condition and each group in three selected time windows. These corresponded to the three phases of beta oscillations modulation calculated as background (between 180 and 625 ms before the stimulus), suppression (between 165 and 378 ms after stimulus) and rebound (between 535 and 980 ms).

The quantitative analysis confirmed that the amplitude of beta oscillations was different as function of time. Indeed, beta oscillations amplitude showed a significant statistical difference in each group and in each condition over the 3 different timing windows (Figure 7).

Repeated measures 2 × 2 × 3 ANOVA with the group [healthy controls vs. patients (ON)], condition (rest vs. movement) and phase (background, suppression and rebound) as factors did not show a significant effect of group [*p* > 0.05; *F*_(1,36)_ = 0.040; Eta^2^ = 0.001]. There was a significant effect of the condition [*p* < 0.05; *F*_(1,36)_ = 34.88; Eta^2^ = 0.493] and a significant interaction between condition and group [*p* < 0.05; *F*_(1,36)_ = 8.739; Eta^2^ = 0.195]. There was a significant effect of the phase [*p* < 0.05; *F*_(1,36)_ = 91.185; Eta^2^ = 0.717]. There was no significant interaction between phase and group [*p* > 0.05; *F*_(1,36)_ = 2.834; Eta^2^ = 0.073]. There was a significant interaction between condition and phase [*p* < 0.05; *F*_(1,36)_ = 15.047; Eta^2^ = 0.295].

*Post-hoc* pairwise comparisons with Bonferroni corrections did not reveal significant difference between the two groups (healthy participants vs. PD ON state) in the rest condition in each phase: background [*p* > 0.05, *t*_(36)_ = 1.090], suppression [*p* > 0.05, *t*_(36)_ = 0.491] and rebound [*p* > 0.05, *t*_(36)_ = 1.235]. The two groups did not show significant difference neither in the movement condition in each phase: background [*p* > 0.05, *t*_(36)_ = −0.645], suppression [*p* > 0.05, *t*_(36)_ = −0.579] and rebound [*p* > 0.05, *t*_(36)_ = −0.370].

Furthermore, *post-hoc* pairwise comparisons showed significant differences between the rest and movement condition in the background phase [*p* < 0.05, *t*_(37)_ = −5.356], suppression [*p* < 0.05, *t*_(37)_ = −4.156] and rebound [*p* < 0.05, *t*_(37)_ = −6.795] over the two groups.

Repeated measures 2 × 2 × 3 ANOVA with the group [healthy controls vs. patients (OFF)], condition (rest vs. movement) and phase (background, suppression and rebound) as factors did not show an effect of the group [*p* > 0.05; *F*_(1,36)_ = 0.0765; Eta^2^ = 0.021]. There was a significant effect of condition [*p* < 0.05; *F*_(1,36)_ = 58.04; Eta^2^ = 0.617] and a significant interaction between condition and group [*p* < 0.05; *F*_(1,36)_ = 7.931; Eta^2^ = 0.181]. There was a significant effect of the phase [*p* < 0.05; *F*_(1,36)_ = 98.454; Eta^2^ = 0.732]. There was no significant interaction between phase and group [*p* > 0.05; *F*_(1,36)_ = 2.366; Eta^2^ = 0.062]. There was a significant interaction between condition and phase [*p* < 0.05; *F*_(1,36)_ = 20.392; Eta^2^ = 0.362].

*Post-hoc* pairwise comparisons with Bonferroni corrections did not reveal significant difference between the two groups (healthy participants vs. PD OFF state) in the rest condition in each phase: background [*p* > 0.05, *t*_(36)_ = 1.446], suppression [*p* > 0.05, *t*_(36)_ = 1.125] and rebound [*p* > 0.05, *t*_(36)_ = 1.725]. The two groups did not show significant difference neither in the movement condition in each phase: background [*p* > 0.05, *t*_(36)_ = 0.112], suppression [*p* > 0.05, *t*_(36)_ = 0.217] and rebound [*p* > 0.05, *t*_(36)_ = 0.484].

Furthermore, *post-hoc* pairwise comparisons showed significant differences between the rest and movement condition in the background phase [*p* < 0.05, *t*_(37)_ = −6.739], suppression [*p* < 0.05, *t*_(37)_ = −5.002] and rebound [*p* < 0.05, *t*_(37)_ = −8.876] over the two groups.

Having found a modulation of beta oscillations amplitude as function of time, the subsequent aim was to test if there was a correlation between beta oscillations amplitude changes across the two conditions and SEPs changes across the two conditions, which was the measure of SA.

This correlation analysis was performed separately for each time window in each group of participants.

**Figure 8:**
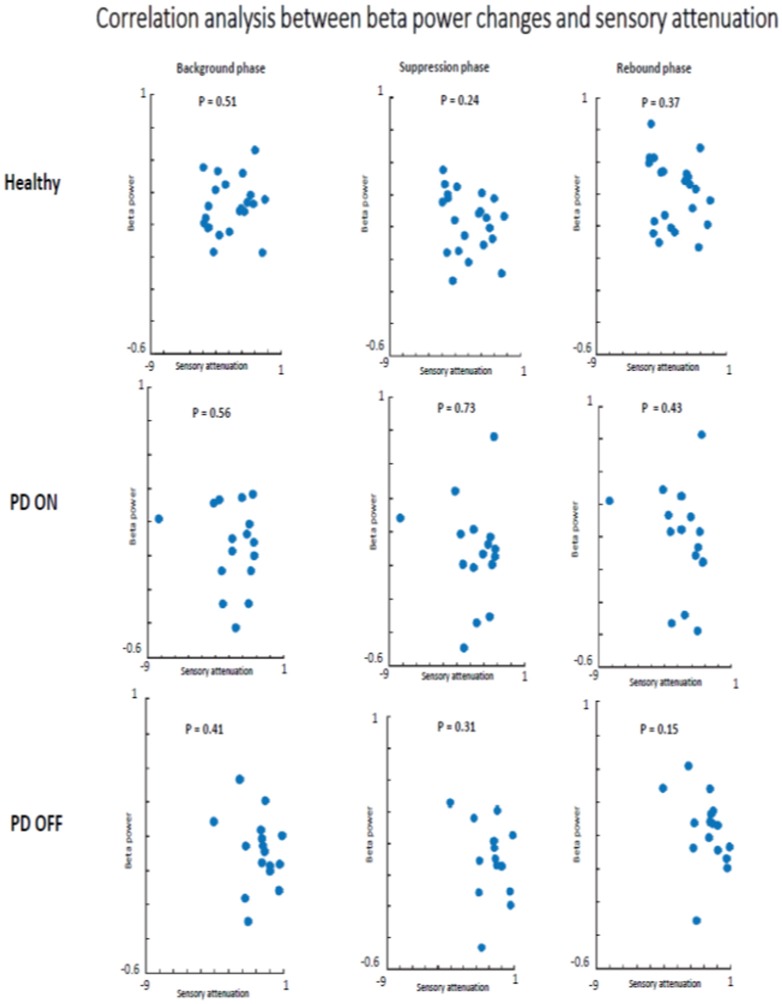
Correlation analysis beta power modulation and sensory attenuation individually in the three groups of participants.

There was no evidence that SA and beta oscillations amplitude modulation were correlated in PD patients ON state and healthy subjects. Indeed, healthy participants did not show a significant correlation between beta oscillations amplitude modulation and SA in background phase (*R*^2^ = 0.04, *p* = 0.51), suppression phase (*R*^2^ = 0.08, *p* = 0.24) or the rebound phase (*R*^2^ = 0.06, *p* = 0.37). The absence of a correlation between these two neurophysiological phenomena was evident also in the PD patients group in ON (background phase, *R*^2^ = 0.11, *p* = 0.56; suppression phase, *R*^2^ = 0.07, *p* = 0.73; rebound phase, *R*^2^ = 0.14, *p* = 0.43) as well as in OFF state (background phase, *R*^2^ = 0.005, *p* = 0.41; suppression phase, *R*^2^ = 0.003, *p* = 0.31; rebound phase, *R*^2^ = 0.006, *p* = 0.15) (Figure 8).

Having not found evidence for a relationship between the degree of SA and the changes in beta power, it was tested if there was a relationship between beta oscillations amplitude and SEPs amplitude. The two measures were measured as a general phenomenon and not as function of the group. Therefore, we investigated if beta oscillations amplitude and SEPs amplitude were correlated in two groups: healthy subjects + PD in OFF state and healthy subjects + PD in ON state.

In the first analyzed group including healthy and PD patients OFF medication, a positive correlation between beta power magnitude and SEPs amplitude was found in the rest condition in all selected time windows (background phase, *p* = 0.02, *R*^2^ = 0.139; suppression phase, *p* = 0.01, *R*^2^ = 0.162; rebound phase, *p* = 0.00, *R*^2^ = 0.220). In other words, lower amplitude of SEPs was correlated with lower beta power amplitude.

**Figure 9:**
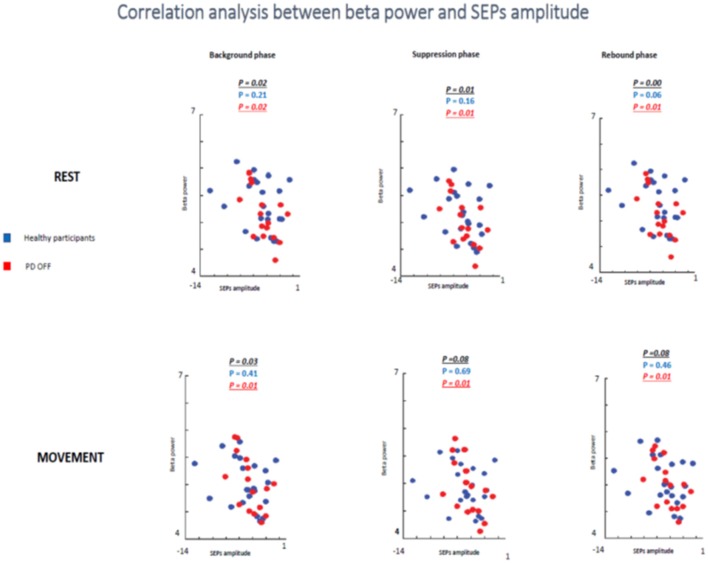
Correlation analysis between beta power and SEPs amplitude at rest and during movements in healthy subjects and PD patients Off medication.

However, this positive correlation seemed to be driven by the PD patients OFF medication. Indeed, when the two groups of participants were analyzed separately, healthy subjects did not show any correlation between beta oscillations amplitude and SEPs amplitude at rest in each time window (background phase, *p* = 0.21, *R*^2^ = 0.07; suppression phase, *p* = 0.16, *R*^2^ = 0.09; rebound phase, *p* = 0.06, *R*^2^ = 0.159). Whereas, the PD OFF medication showed a significant correlation between the two measures at rest in all time windows (background phase, *p* = 0.02, *R*^2^ = 0.304; suppression phase, *p* = 0.01, *R*^2^ = 0.335; rebound phase, *p* = 0.01, *R*^2^ = 0.371) (Figure 9).

In the movement condition the group including healthy subjects and PD OFF patients still showed a significant correlation between the two conditions in the background timing window (*p* = 0.03, *R*^2^ = 0.113) and a statistical trend in the suppression phase (*p* = 0.08, *R*^2^ = 0.08) and in the rebound phase (*p* = 0.08, *R*^2^ = 0.08). Interestingly, this correlation was driven by the PD OFF patients. Indeed, when the two groups of participants were analyzed separately the significant correlation was kept only by PD OFF medication. The control group did not show any correlation in all time windows (background phase, *p* = 0.41, *R*^2^ = 0.03; suppression phase, *p* = 0.69, *R*^2^ = 0.008; rebound phase, *p* = 0.46, *R*^2^ = 0.02), whereas PD OFF medication showed significant correlation between beta oscillations modulations and SA in the three time windows (background phase, *p* = 0.01, *R*^2^ = 0.363; suppression phase, *p* = 0.01, *R*^2^ = 0.351; rebound phase, *p* = 0.01, *R*^2^ = 0.354).

In the second analyzed group including healthy participants and PD patients ON medication a statistical trend of the correlation between beta oscillations amplitude and SEPs amplitude was found in the first two times windows (background phase, *p* = 0.09, *R*^2^ = 0.07; suppression phase, *p* = 0.07, *R*^2^ = 0.08) and a significant correlation in the rebound window in the rest condition (*p* = 0.01, *R*^2^ = 0.144). However, it is likely that this result was driven by the power of this bigger sample.

**Figure 10:**
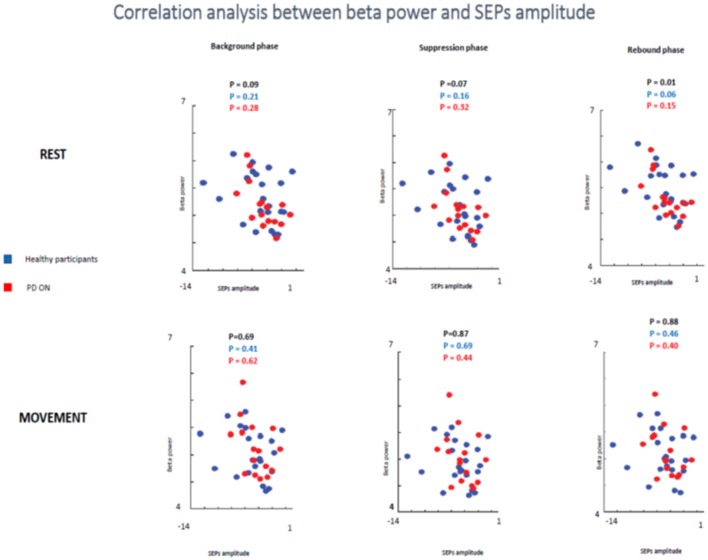
Correlation analysis between beta power and SEPs amplitude at rest and during movements in healthy subjects and PD patients ON medication.

When the two groups of participants were analyzed separately, neither groups showed any significant correlations between the two measures in the rest condition in any time windows. Healthy subjects did not show a significant correlation in the background phase (*p* = 0.21, *R*^2^ = 0.07) or in the suppression phase (*p* = 0.16, *R*^2^ = 0.09). There was a statistical trend in the rebound window (*p* = 0.06, *R*^2^ = 0.159). PD patients ON medication did not show significant correlation in background phase (*p* = 0.62, *R*^2^ = 0.08), suppression phase (*p* = 0.44, *R*^2^ = 0.06) and rebound phase (*p* = 0.40, *R*^2^ = 0.137) (Figure 10).

In the movement condition, there was no significant correlation in all analysis (healthy participants + PD ON patients and separately healthy subjects and PD ON). The combination of healthy controls and PD patients in ON state showed the following results: background phase, *p* = 0.69, *R*^2^ = 0.04; suppression phase, *p* = 0.87, *R*^2^ = 0.001; rebound window in the rest condition, *p* = 0.88, *R*^2^ = 0.001).

When the two groups of participants were analyzed separately, neither groups showed any significant correlations between the two measures in the rest condition in any time windows. Healthy subjects' group did not show a significant correlation in the background phase (*p* = 0.41, *R*^2^ = 0.034) or in the suppression phase (*p* = 0.69, *R*^2^ = 0.008). There was a statistical trend in the rebound window (*p* = 0.46, *R*^2^ = 0.027). PD patients ON medication did not show significant correlation in background phase (*p* = 0.62, *R*^2^ = 0.017), suppression phase (*p* = 0.44, *R*^2^ = 0.043) and rebound phase (*p* = 0.40, *R*^2^ = 0.050) (Figure 10).

These results might be explainable by the presence of SEPs attenuation in both groups at the onset of the movement. Therefore, SEPs amplitude was lower at the onset of the movement compared to the magnitude at rest but beta does not change as function of condition, therefore the correlation was not significant.

## Discussion

These results confirmed our previous study (8). A significant link was found between dopaminergic modulation and SA. Indeed, at movement onset PD patients off medication showed a lower SA compared to PD patients ON medication. The mean difference of the N20-P25 amplitude between rest condition and movement condition was significantly different between PD patients OFF medication and ON medication.

This result of lower SA in PD patients OFF medication is in line with previous studies that have shown abnormal SA in PD (19, 20). It is important to consider that there were critical differences in the task design. In previous studies (19, 20), patients were tested making vigorous wrist flexion and extension movements. In addition, SEPs were recorded during continuous movement. In our study, subjects performed a movement of the thumb and the median nerve stimuli was delivered at the onset of the voluntary movement. Our results supported the hypothesis that a failure in SA prior to movement onset contributes to the difficulties in movement initiation in PD.

In line with previous studies (12, 21), healthy subjects showed changes in beta oscillations as attenuated prior the voluntary movement and augmented once the movement has ended.

A potential correlation of SA with cortical beta oscillations in the cohort of PD patients and age-matched healthy subjects was hypothesized. The study was focused in understanding the functional role of beta oscillations as it is well-known that PD patients have a pathologically higher power of beta oscillations, both in the cortex (16) and sub-cortically in the subthalamic nucleus (16, 22–24). Of note, levodopa treatment (22, 23) and subthalamic deep brain stimulation for PD (22, 23, 25, 26) are associated with a decrease in beta power. On the other hand, it is well known that stimulation of the subthalamic nucleus at the beta frequency (15–30 Hz) causes a slowing of movement in patients with PD (27). Consequentially, the high amplitude of beta oscillations in PD was proposed as a cause of bradykinesia (16). However, the mechanism underlying this hypothesis is still not clear.

This study provided evidence that physiological SA could be the neurophysiological mechanism underlying the bradykinesia. Therefore, if both these mechanisms (physiological SA and high beta oscillations) have been hypothesized as underlying the bradykinesia, a correlation between these two mechanisms was proposed. Specifically, it was tested whether the modulation of SA was correlated with the modulation of beta oscillations during voluntary movements.

Our results did not show significant evidence of a modulation of cortical beta oscillations driven by the sensory-motor cortex on SA. This finding can be interpreted in two ways, either that the cortical beta oscillations are not involved in modulation of SA or that our groups' size was not enough to reach the statistical power.

Regarding the first possibility, although there is no direct evidence of a potential link between cortical beta oscillations and SA, it is known that the beta oscillations plays important role on the modulation of motor control. In particular, it has been shown that the modulation of beta oscillations shows a particular pattern during voluntary movements (12). This modulation of beta oscillations takes place at the onset of voluntary movement, when SA is also present. Consequentially, there is a rationale to explore if the SA modulation is correlated with beta oscillations modulation over the sensorimotor cortex. On the other hand, the beta oscillations are present not only at the cortical level but also at the subcortical level as in the basal ganglia, which were not explored in this study. From the above, it was not possible to determine whether or not subcortical beta oscillations play a modulatory role on SA. In order to address this issue further, it would be necessary to investigate SA in PD patients with STN-DBS to test if there is a correlation with the abnormal beta oscillations in STN, typically seen in this group of patients.

Regarding the second possibility, it is well known that a major fault of scientific studies (including ours) is inadequate statistical power. A larger number of subjects were required to adequate power the studies because of increased variability of SA as well as cortical beta oscillations in the patient population. Although this is a major limitation for any conclusion about the mean of potential link between SA and beta oscillations in patients with PD, the fact that SA was replicated to be reduced in patients with PD OFF dopaminergic treatment is noteworthy on its own. Increased variability may have important implications in the design and interpretation of future studies and may indeed be related to pathophysiological mechanisms of PD.

The results of this study did not support the theory suggesting that the modulation of physiological SA and modulation of beta oscillations over the sensorimotor cortex are related. However, it was confirmed the modulation of the two parameters during voluntary movements. In particular, the two groups of participants showed reduced beta power just prior to and during the period of movement and transiently increased subsequent to the end of the movement. This result is in line with previous studies (28, 29). Furthermore, several studies showed evidences that beta oscillations play a role in sensorimotor processing (30–33). In this regard, Baker et al. (31) found that beta frequency showed a coherence between proprioceptive afferents (Ia muscle spindles) and forearm muscle activity, suggesting that beta oscillations may have a role mainly in proprioceptive processing. On the contrary, there was no coherence between muscle activity and afferents relate to cutaneous receptors. However, Witham et al. (33) did not find a difference in coherence with M1 between areas 1 and 3b, which are associated to cutaneous receptive fields, and areas 3a and 2, which are associated with proprioception (areas 3a and 2). Therefore, this study provided evidence for a close link between the sensory and motor systems via oscillatory synchronization and support previous hypotheses that this pattern of activity may be important in coordinating the processing of somatosensory information within its motor context (32, 33).

The current study did not confirm a role of beta pattern activity in coordinating the somatosensory integration at least in terms of SA.

This study did not show a significant different amplitude in the cortical beta oscillations between PD ON and healthy controls as well as between PD OFF and healthy controls. Therefore, these results bring under discussion the pathological role of sensorimotor beta oscillations in PD. There is a need to be a replication of the study on a larger group of PD patients to confirm these results. Additionally, further studies are needs to test a potential correlation between physiological SA and beta oscillations generated in the basal ganglia with the aim to test if modulation of SA is correlated with this other pattern of beta activity.

## Data Availability

The raw data supporting the conclusions of this manuscript will be made available by the authors, without undue reservation, to any qualified researcher.

## Ethics Statement

The study was approved by the local institutional ethics committee, which was the East of Scotland Research Ethics Service. Written informed consent was obtained from all participants.

## Author Contributions

AM: design of the study, collecting data, EEG analysis, statistical analysis, writing manuscript. PL, PK, TF, and ME: design of the study, reviewing of the manuscript. JK: design of the study, EEG analysis, statistical analysis, reviewing manuscript.

### Conflict of Interest Statement

The authors declare that the research was conducted in the absence of any commercial or financial relationships that could be construed as a potential conflict of interest.
